# Being the Father of a Preterm-Born Child: Contemporary Research and Recommendations for NICU Staff

**DOI:** 10.3389/fped.2021.724992

**Published:** 2021-09-06

**Authors:** Franco Baldoni, Gina Ancora, Jos M. Latour

**Affiliations:** ^1^Department of Psychology, University of Bologna, Bologna, Italy; ^2^Neonatal Intensive Care Unit, Infermi Hospital, Rimini, Italy; ^3^Faculty of Health, School of Nursing and Midwifery, University of Plymouth, Plymouth, United Kingdom; ^4^Department of Nursing, Hunan Children's Hospital, Changsha, China

**Keywords:** father, preterm infants, family-centered care, neonatal intensive care unit, perinatal affective disorders, COVID-19

## Abstract

**Background:** Most studies on parental reactions to a preterm birth and to hospitalization of the newborn in Neonatal Intensive Care Units (NICUs) have involved mothers. However, emotional responses and behaviors of fathers are equally important. Usually, the father is the first to meet the preterm newborn, to find out information about baby's condition and to communicate to the mother and other family members. In this context he is often left alone and can show psychological difficulties including affective disorders such as depression or anxiety. This paper describes the role of fathers in the NICU, the best practices to support fathers, and to explain the role of a psychologist in the NICU staff. Considerations and suggestions are provided on the difficulties encountered to support parents, with a focus on the role of fathers during the COVID-19 pandemic.

**Methods and Discussion:** Considering contemporary research data and following an attachment perspective, we analyze the role of the father of a preterm-born child in the relation with the partner and in newborn caring. Research has shown that involving fathers in newborn care in NICU and at home is essential not only because it promotes the father/son attachment relationship and has positive effects on the psychological and somatic development of the newborn, but also for the health of the mother and whole family.

**Conclusion:** Recommendations are provided to enhance the functions of fathers in the NICU, promote their involvement in the care of their infant, and interventions to prevent the manifestation of psychological suffering and/or perinatal affective disorders. The commitments of a psychologist in a NICU team are presented and require not only clinical skills, but also the ability to manage the emotional and relational difficulties of fathers, family and NICU staff. Considerations and suggestions are provided on the difficulties encountered by parents in the NICU during the COVID-19 pandemic.

## Introduction

**Figure 1:**
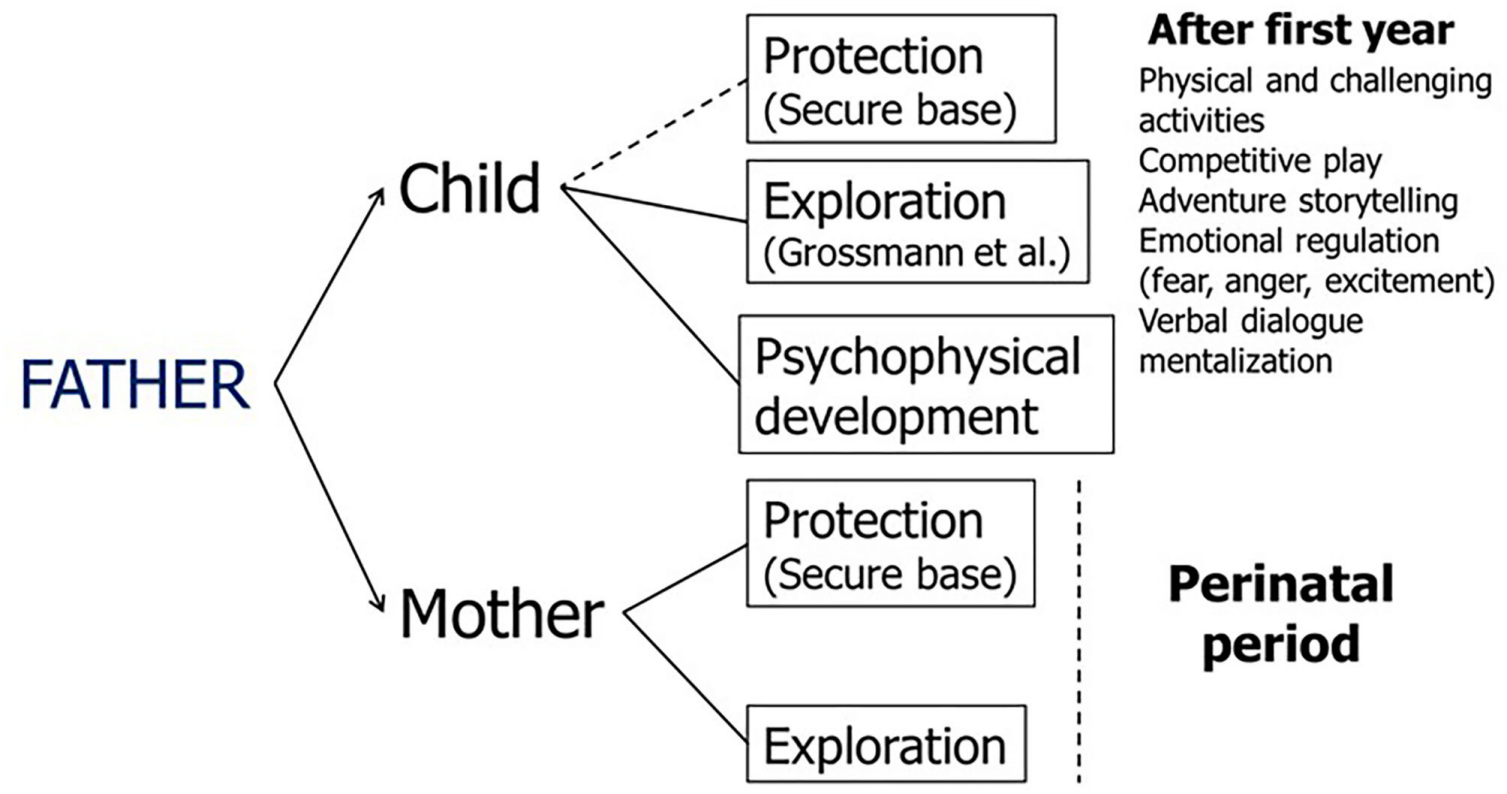
Paternal attachment functions.

In recent years, research has provided convincing evidence on the importance of fathers since the first moments of pregnancy and its influence on mother-child relationship, mental health of mothers and the psychophysical development of infants. The attachment between father and son is now considered more important than previously thought, alongside the attachment theory on the function of the mother as attachment figure (1). The aim of this paper is to describe the role of fathers in the Neonatal Intensive Care Unit (NICU), the best practices to support fathers in the NICU, and to explain the role of a psychologist in the NICU staff. Considerations and suggestions are provided on the difficulties encountered by parents, with a focus on the role of fathers in the NICU during the COVID-19 pandemic.

## Family Attachment and Function of Fathers in the Perinatal Period

In the last decade, research has demonstrated that the male is on a natural biological evolution when caring for children, which is evidenced by hormonal and neurobiological changes that occur when fathers care for newborns (2–7). These biological changes are related to increased levels of *oxytocin* (which favors empathic abilities, social activities, and willingness to play) (8, 9), the decrease in *testosterone and estradiol* (which makes fathers more sensitive, less aggressive and better disposed toward newborn and mother) (10–13), higher levels of *prolactin* (which increases when the baby cries or is more vulnerable and in need of care) (14, 15), *vasopressin* (which in animals favors the territoriality and protection of the partner) and *cortisol*, a classic stress hormone (which intensifies attention toward the newborn, but which decreases during the “Skin to skin” contact) (8, 14). A higher prenatal level of cortisol, however, is predictive of a lower quality of postnatal parenting of fathers (16).

The brain areas and circuits activated when caring for a newborn are similar in men and women and concern emotional-empathic and socio-cognitive brain functions (2, 5, 6). Therefore, similarly to the mother, the father is also biologically predisposed to an early attachment relationship and this relationship plays a role in the child's psychophysical development. Over time, as the child grows, the influence of father-child attachment is linked not only to the father's ability to speak to him and think of him but also in telling stories and to involve him in physical and exciting activities, competitive sports and games (1, 17, 18). These experiences will be decisive for the development in children of a valid regulation of emotions and impulses, particularly aggressive ones, and of capacities which are in turn mentalizing (reflective). They will also encourage them to explore the family and extra-family environment (19).

The father's emotional-empathic brain activities at 1 year of life of the infant favors the development of a better emotional regulation of the child at 4 years of age, while socio-cognitive ones favor social skills (9). These will be very useful in managing extra-family relationships (between peers, with school, with the first sentimental partners), especially during adolescence and the period of autonomy of the children. It would be an understatement, however, to consider the father only in the direct relationship with the son (the same applies to the mother). In fact, one of his fundamental tasks during pregnancy and infancy of the offspring is to guarantee the conditions for the relationship between mother and baby to develop and be adequately maintained (20). In the first place, the father must deal with practical problems: ensuring a comfortable and safe home, providing financial support, food, and other necessary goods, relating to the extrafamilial environment, protecting the family and solving any problems and conflicts.

During the perinatal period, women are exposed to emotional alterations and psychological difficulties favored not only by physical changes, but also by their role as becoming a mother. An important function of the father, in these cases, is to act as an attachment figure and help his partner to overcome the difficulties by keeping concerns and suffering at acceptable levels. This is often done by providing security and emotional support, encouraging the partner to her new maternal role, and protecting her from an excess of psychological suffering (21). In the perspective of attachment theory, this protective function can be interpreted as a “secure base” effect (20, 22, 23), which is the result of the atmosphere of security and trust that characterizes the couple attachment. This secure base function is particularly important in the case of a high stressful event like a pre-term birth. Therefore, during pregnancy and after the birth of a child, a partner who is too worried, anxious or depressed, represents a disadvantage for the mother.

The paternal attachment functions are summarized in Figure 1 according to the current evidence (1, 17–19). In the first months of life, the paternal protective function toward the child is less important than the maternal one, which is more exposed in body contact with the newborn (24), but intensifies from the second year of life, favoring exploration of the environment, emotional regulation and the development of mentalizing and relational skills (through verbal dialogue, the telling of exciting stories, physical activities, adventurous, and competitive play). During the perinatal period, an important function of the father is carried out toward the mother, who must be protected from the physical and emotional difficulties of pregnancy and supported in the exploration of the new condition of motherhood.

## Being the Father of a Preterm Born Baby

The preterm birth of a child is an unexpected event that profoundly influences personal and family life in parenting, couple's relationship and that with other family members, especially grandparents and siblings of the newborn. The premature infant is born when parents are “psychologically premature” (25) and still cannot perceive it as distinct and separate from the mother. It is a threatening event that imposes the need for hospitalization in the neonatal intensive care unit (NICU), causing a condition of stress and concern in which not only the health and life of the infant is in danger, but also the emotional balance of the family members (4, 20, 26, 27). The experiences of the NICU period creates strong emotional reactions in parents. The most common are (20, 24, 28):

- *Fear*: regarding the foreign and medical environment and the child, who can become seriously ill, disabled, or die.
- *Anger*: for the failed expectations regarding the birth, the inability to control the events or for family, friends, partner, or oneself.
- *Blame and shame*: “What did I do to deserve this?,” “Where did I go wrong?,” “If only I had done….”
- *Sensations of loss*: regarding expectations and future, one's identity as a normal parent.
- *Envy*: toward other parents who have a term born healthy child.
- *Impotence*: many other people are taking care of your child, you would like to be consoled and helped, but you don't know who to turn to.
- *Feeling under everyone's eyes*: large common areas, no privacy, no possibility of confidentiality and private expression of feelings. You feel like “a fish in an aquarium.”

In this anguished and threatening situation in the NICU, the attachment system whose function is protection from danger is activated (29). Therefore, defensive and potentially problematic behaviors can occur in both parents, limiting the desire to establish emotional contact with the premature infant (and the partner) and negatively influencing couple life and the development of parent-child attachment. In this situation parents' needs are to be protected, reassured, informed, and guided. However, the way to express these needs depends on their attachment experiences (past attachment relationships and possible exposures to danger) and on the quality of their couple and family relationships (20). On the other hand, the responses of the healthcare staff are also influenced by the attachment experiences of doctors and nurses.

Most studies on reactions to a preterm birth have concerned mothers, and the same is true for the modalities of care and interventions (26). But the emotional responses and behaviors of the father in these cases are equally important (7). Usually, the father is the first person to see the hospitalized infant. It is up to the father to gather information on the condition of the infant and the first care provided. He must then communicate these information to the mother and other family members, acting as the spokesperson and interpreter of the healthcare workers. If there are siblings, the father must take care of them too. In these functions he is often left alone, despite himself being in a state of concern and disorientation. The difficulties manifested by these fathers in the relationship with their preterm-born baby has been documented in a study where fathers showed high levels of anxiety, depression, and stress (30). Regardless of term or preterm birth, these characteristics were related, both in fathers and mothers, to a lower psychomotor development of the newborn, assessed at 6 months corrected age through Bayley scales (*p* = 0.007). In the case of a preterm birth, the father's uneasy condition tends to continue throughout the hospitalization and, often, intensifies after discharge from the hospital, when the parents, after a more or less long period of specialist assistance, find themselves having to manage a newborn at home who continues to need constant medical attention. This is the period of greatest difficulty.

Having a preterm born baby affects the quality of life (QoL) of the couple and the family (31). A recent cross-sectional study (32) on parents of very preterm infants showed that parental stress, anxiety and depression symptoms negatively influence both maternal and paternal QoL. Moreover, the impact of socioeconomic position and infant-related factors varies according to gender and the domain of QoL. For example, fathers of infants hospitalized for 2 months or more and with health problems presented lower levels of social QoL. Infant-related factors such as NICU length of stay, health problems and extremely low birth weight were associated with the physical, psychological, social, and environment domains of QoL among fathers. This information is useful for the implementation and integration of family-centered care in neonatal healthcare settings.

Often parents worry about each other (33). Fathers tend to worry about mothers' emotional conditions, mothers about their partner's working difficulties (34). In the long run, hospitalization and medical expenses can lead to serious financial problems with consequences on the quality of food and personal care, on the choice of a home, on social relationships and leisure time (27). Economic difficulties and lower quality of life can lead to further social and health problems and the impact is greater if the child has a severe handicap or serious intellectual deficit. Already during the hospitalization, the attention on the child is so high that many parents forget their personal and couple needs. For this reason, the experience of a preterm birth and hospitalization in NICU, if not well-supported, can fuel couple conflicts and lead to separation or divorce. A Danish study including 959 parents of preterm children found that, after 19 years after a NICU experiences, the divorce rate was much higher than in the general population, especially if the child was disabled, and 36% of mothers had had negative consequences on the job (35).

The frustration of personal needs, increase in responsibilities, couple difficulties, working difficulties, and limitations of freedom while caring for a child entail an increased risk of affective disorders for both parents, in particular depression and anxiety disorders. These problems in fathers are very frequent but tend to be underestimated.

## Paternal Perinatal Affective Disorders

For decades, research into perinatal affective disorders (particularly postpartum depression) has focused almost exclusively on mothers. The reasons are different (20, 36):

- The function of the father is often underestimated by the healthcare professionals, who consider pregnancy and childbirth as predominantly a female problem (37). This attitude, named *maternal gatekeeping* (38), tends to exclude fathers or to legitimize their disengagement and is often favored and shared by mothers.
- Fathers show a lack of willingness to be engaged in clinical studies and are often reluctant to ask for help and to communicate their problems, especially psychological ones.
- Men tend to manifest their difficulties, in particular affective ones, in a different way than women, minimizing the depressive aspects. Consequently, paternal affective disorders tend to be considered clinically less serious.
- Self-report tools designed to assess female depressive symptoms and that do not take gender differences into account are usually used for screening and diagnosis (36, 39, 40).

Despite these difficulties, recent systematic reviews and metanalysis have demonstrated that in the perinatal period fathers frequently experience affective disorders almost as much as mothers. In fact, about 13% of women suffer from perinatal affective disorders, and 8.4% to over 10% of men have the same problem (41–43). Furthermore, about 50% of males with a partner suffering from post-partum depression is depressed, as well as 50% of mothers with a partner who has a perinatal affective disorder suffers from the same disease. Throughout the period before and after pregnancy, the mental states of father and mother influence each other and this increases the risk of a psychopathological disorder when the partner also suffers from it (41–43). Paternal perinatal affective disorders, such as Paternal PeriNatal Depression (PPND), are therefore very frequent, but their diagnosis is difficult. In men, depressive symptoms tend to manifest themselves differently from mothers and are frequently accompanied by other disorders (36):

- *Anxiety disorders*: general anxiety disorders, panic attacks, phobias, post-traumatic stress disorders), which affect up to 18% of fathers.
- *Abnormal illness behavior*: somatization disorders, functional medical syndromes, hypochondriac concerns.
- *Behavioral disorders*: anger attacks, violence, compulsive physical or sexual activity, extramarital affairs, and escapes from home or at work.
- *Substance abuse and other addictions*: smoking, alcohol, drugs, gambling, compulsive use of smartphone or social networks, and internet addiction disorder.

These disorders, which can overlap the depressive symptomatology or mask it, generating complex and difficult to define clinical pictures, tend to occur more frequently in the third trimester of pregnancy and 3–4 months after delivery, with a frequency almost three times greater than in the general population (44). Recognizing the manifestation of an affective disorder early is very important as it has negative consequences not only for the father, but also for the well-being of the mother and for the development of the child; risks increase in the case of a preterm birth (36).

## What the Multidisciplinary Staff, Including a Psychologist, Can Do?

Research have demonstrated that involving fathers in the care of newborns is essential not only because it promotes attachment between father and son and has positive effects on the development of the newborn, but also for the health of the mother and of the whole family (45). These results have sensitized gynecologists, pediatricians, neonatologists, and nurses to the need to support both parents, since the beginning of pregnancy, in playing an active role in the care of their baby, in the context of family-centered care. This is particularly important in the NICUs where the need to resort to specialized medical equipment can make parental involvement very difficult. Considering the current evidence supporting the extraordinary importance of the early relationship with the newborn and contact with both the mother and the father, it is necessary, as indicated in all the guidelines in this area, to encourage the presence in the ward of both parents by opening it for 24-h visits (4).

As for the direct body contact of the father with the newborn, facilitated by practices such as tactile stimulation or Kangaroo care, it has been shown that, similarly to that with the maternal body, it performs beneficial functions on oxygenation of blood (46), frequency of breastfeeding (47), body temperature, blood glucose levels, decrease in salivary cortisol levels and on the tranquility of the newborn (48). Also, it stimulates the father to take care of the child by promoting the development of an attachment bond (49). Promoting the presence of the father in the NICU (4, 37) and offering family-centered care (or family integrated care), which considers both parents, favors the weight gain and breastfeeding (50, 51) and reduces the separation time of the infant from the parents (52).

The Family Initiative's International Neonatal Fathers Working Group (4), an international group of researchers who has been working on the presence of the father in the NICUs for years, has identified three basic principles to follow:

1) Support the father-child bond in the same way as that with the mother.
2) Pay attention to the differences between mother and father, both in terms of families of origin and social expectations regarding female and male roles.
3) Support co-parenting by mother and father.

More specifically, to enhance the functions of fathers of preterm babies, promote their involvement and prevent the manifestation of perinatal affective disorders and couple problems, the following guidance needs to be considered in clinical practice (4, 7, 36, 39):

1) **Consider the father from the first moment of admission to NICU, recognizing his role in the couple and in the family, informing him, advising, and supporting him psychologically**. It is necessary to speak with the father not only in the presence of the mother or through the mother, but also individually, giving him the opportunity to express his experiences without feeling judged, free to let himself go in conditions of confidentiality and security. He should also be helped to regulate emotional tension without feeling too out of control (53). Given the importance of the direct relationship between father and infant, it is necessary to encourage moments of exclusive and reserved contact with the baby (54), including tactile stimulation or Kangaroo care. Help the father recognize how the infant reacts to his presence and how he calms down when the father takes care of the infant (55). Information about the infant and the correct behavior to be followed in the relationship with the father must be directed to both parents in a co-parenting perspective. It is important to speak of the newborn child not as a “patient,” but as a child who arouses emotions and feelings, who has his own temperament, personality and future.
2) **Encourage the father's relationship with the other fathers of preterm born children who attend the ward**. This not only helps to feel less alone, to share difficulties with others who are in the same situation and to exchange opinions and advice, but also limits the feelings of envy toward parents who have had a child born without problems. Today, the fathers of preterm born children also communicate with each other through social media and by attending special forums on websites (46).
3) **Fathers often exhibit affective disorders, but differently from women**. In particular, depressive suffering can be masked by externalizing symptoms or problematic behaviors (anxious or somatic complaints, anger attacks, and aggressive behavior, abuse of smoking, alcohol and other addictions). To assess these emotional difficulties, it is necessary to consider gender differences and use methods and tools that take into account the different expression of affective disorders in the male (36). Self-reported tools such as the Edinburgh Postnatal Depression Scale (EPDS) (56) may be used for screening, referring to cut-off scores identified for the fathers (57) or using the Perinatal Assessment of Paternal Affectivity (PAPA) (58), one of the few that evaluates not only the anxious and depressive symptomatology, but also the other ways of expressing the emotional suffering of fathers. For a correct prevention of male affective disorders and their consequences on the health of the mother and newborn, the father, however, should be considered from the beginning of pregnancy, involving him in gynecological visits and in family counseling activities. In the specific case of a preterm birth, both parents should be assessed psychologically at least twice: during the first week of admission to NICU and before discharge (4).
4) **Consider the possible manifestation of an affective symptomatology both in the mother and in the father**. When one parent is depressed, the possibility that the other also suffers from a similar disorder must be carefully considered (59– 61). In such cases, the negative effects on the development of offspring increases (62). The risk of maternal and paternal depression is greater when the infant is perceived by both parents as “problematic” (63) on in the case of a preterm born child (30).
5) **The problem of preterm birth must be assessed within a systemic family perspective, considering not only the parent-child dyad, but also the parental couple and the mother-father-child triad**. Often there is a deterioration in married life, with misunderstandings, conflicts and quarrels. It may be necessary to provide the parents with the opportunity to discuss their problems by helping them improve their relationship or resolve a couple crisis.
**6) To inform fathers about the difficulties they may encounter in the relationship with their preterm born child and about the psychological and relational problems that may affect them, their partner and the couple's life**. Mothers should also be informed about the possibility that their partner may feel in difficulty and about the specific ways in which men express their need for help (64). The help from the partner is essential to limit the psychological suffering of the parent and to encourage his/her adaptation to the life of the ward and to the specific needs of the newborn.
7) **NICU staff must be prepared to relate to the whole family and to recognize the early signs of a paternal perinatal affective disorder**. Training courses or seminars on this topic can raise awareness among healthcare professionals and promote the screening and prevention. Nurses play an important role in involving fathers (65). To encourage the development of a psychological sensitivity and greater attention in the operators toward relationship dynamics, strategies need to be implemented that helps the NICU staff to pay attention to the emotional and relational aspects of family members who attend the ward (66).
8) **The support provided to the family, including grandparents and siblings, must continue after discharge**. It is useful to organize periodic home visiting interventions, during which information can be given, but also advice on what attitude to adopt toward the newborn. During the visit, video-feedback techniques based on the audio-video recording of the interaction between parents and child can be used (36). Parents, through viewing the videos and with the help of the clinician, can enhance their skills, but also recognize the difficulties and inadequate ways of relating. These methods, of which there are several models, refer to the paradigms of attachment, infant research and developmental psychology and have been useful for promoting sensitivity and mentalization skills of parents. These interventions have also been used successfully during the hospitalization of the newborn in NICU (67). Following a perspective of both dyadic and triadic intervention, it is possible to involve the mother and the father, together or individually. In fathers who have a partner suffering from postpartum depression, they have produced positive effects both on the relationship between parents and child and on that of the couple (68).

The role of the psychologist in the NICU is very delicate and requires specific expertise and experience. An inadequate psychological intervention toward a family exposed to a potentially traumatic experience, in fact, can be worse. For this it is necessary that the psychologist is a permanent member of the NICU staff and not an external or occasional consultant (66, 69).

Within the NICU workforce, today the inclusion of a psychologist has increased the benefits to parents and some departments have a specialist psychologist within their staff (4, 7). Often, they are consulted to talk to parents, listening to them, helping them to accept the situation and deal with it without feeling alone. The risk, however, is that of being delegated the responsibility in the management of parents by colleagues, especially in problematic situations such as anger, despair, or crying). Therefore, whenever possible, interviews with parents should be conducted collaboratively with a doctor or a nurse. In fact, NICU doctors and nurses are the most exposed in the relationship with the family and must be prepared to support their emotional load and be of assistance in critical moments. Supporting colleagues is therefore one of the most important functions of a psychologist in the NICU. Specific team meetings can be organized to help staff to manage their emotional and relational difficulties. The psychologist can help colleagues to recognize and interpret the emotional reactions of mothers and fathers, but also communicate their experiences with them, analyzing the relationship dynamics and evaluating the most appropriate responses to the situation.

To conclude, the functions of a psychologist, in the case of a preterm birth, are complex and require not only clinical skills, but also psychological balance and ability to manage the emotional and relational difficulties not only of the parents, but also of colleagues. Unfortunately, NICUs do not always have a psychologist among their staff and more often resort to the advice of colleagues outside the department. Psychologists who work with the families of preterm babies have rarely followed specific training courses, but usually rely on the experience gained within the ward. The psychological training of all healthcare professionals who work with the families of preterm babies and improvement of their ability to involve the father in the care of the newborn, are challenges that will need to be addressed.

## Fathers in NICU During COVID-19 Emergency

During the COVID-19 pandemic, practices within many NICU has profoundly changed. Access by parents to their infant and the NICU has been limited. In our experience, to respect social distancing in the space limited areas of NICU, parents are alternating their daily unrestricted presence in NICU, wearing face mask; siblings have instead restricted access to the unit. This new organizational model has brought challenges and opportunities in performing family-centered care practices during the COVID-19 pandemic. The first challenge seems to be the communicative one; indeed, missing facial expressions because of facial masks, makes it difficult to modulate verbal communication among parents and professionals and to interpreter parent's reaction to communication. Moreover, at the bedside, parents are alone in communicating with staff, without the support of the partner, feeling the emotional burden of reporting updates about the baby to the whole family. We believe that some of the emotional difficulties emerged during this period are related or reinforced by the impossibility for parents to stay together and support each other during the NICU stay, thus reinforcing the importance of the co-regulation in NICU between parents, among parents and newborn and among the parents of newborn and staff.

We have, nevertheless, tried to pursue a family-centered care perspective reinforcing parents' empowerment and planning weekly multidisciplinary meetings with both parents, according to their schedule, especially taking into account the fathers work commitments, including a neonatologist, nurse, psychologist, Newborn Individualized Developmental Care and Assessment Program (NIDCAP) professional, physical therapist and cultural mediator or other specialist, when appropriate. Non-structured interviews with fathers were also performed by a NIDCAP professional in order to explore father's feelings, as we believe that fathers are those mostly hampered by the restrictive visiting policies, although their role in providing emotional support to mothers is well-recognized. With this approach, in spite of the pandemic challenges, all the mothers of the very low birth weight infants, and about 75% of the fathers, practiced early and unrestricted skin-to-skin contact (SSC) with their babies during the first COVID-19 pandemic lockdown. Moreover, all fathers and mothers performed daily care of their babies (i.e., tube feeding, mouth care, and nappy change). Father's interviews unveiled a loving engagement with their babies as mentioned: “At the beginning I was loath to touch the baby, but now I enjoy physical contact with him”; “I am also able to manage her nasal prongs”; “Now, I'd like to stay always in SSC, because it gives me a sense of safety and helps me to prepare to go home with him”; “Taking care of him helps me to be in tune with nurses”; “I'm happy to stay in SSC with my daughter, if I could I'll do it continuously. I love to give her a delicate massage behind the ear.” During the COVID-19 pandemic, we observed that fathers, without the mother co-presence, took up the challenge of caring for their babies as primary caregivers. Also, nurturing staff has helped staff to nurture parents, giving, especially to fathers, the opportunity to establish an intimate contact with their preterm born baby, improving the confidence in their paternal role, and as primary caregivers and attachment figure during this difficult period.

## Summary

Most studies on parental reactions to a preterm birth and NICU admission have involved mothers. Unfortunately, the emotional responses and needs of father has been underrepresented in the current evidence base. The father is often the first point of contact in the NICU. In this context he is often left alone and can show psychological difficulties including affective disorders such as depression or anxiety. Using contemporary research data this paper provides suggestions to improve the support of fathers and increase their confidence in their role in the NICU. Involving fathers in newborn care in NICU and at home is essential. Not only because it promotes the father/son attachment relationship and has positive effects on the psychological and somatic development of the newborn, but also for the health of the mother and other family members. The suggestions provide directions for healthcare professionals to enhance the functions of fathers in the NICU, promote their involvement in the care of their infant, and interventions to prevent the manifestation of psychological suffering and/or perinatal affective disorders. Including a psychologist in the NICU team can benefit the fathers, mothers, and the NICU team to work collaboratively to a healthy environment in the NICU that benefit the infant (long-term) outcomes.

## Author Contributions

FB, GA, and JL initiated the review of the topic and contributed to the development of the review, the suggestions, and recommendations. FB and GA drafted the first manuscript. All authors contributed to manuscript revisions, approved the final version of the manuscript, and agreed to be accountable for the content of the work.

## Conflict of Interest

The authors declare that the research was conducted in the absence of any commercial or financial relationships that could be construed as a potential conflict of interest.

## Publisher's Note

All claims expressed in this article are solely those of the authors and do not necessarily represent those of their affiliated organizations, or those of the publisher, the editors and the reviewers. Any product that may be evaluated in this article, or claim that may be made by its manufacturer, is not guaranteed or endorsed by the publisher.
